# Cognitive and Emotional Mapping With SEEG

**DOI:** 10.3389/fneur.2021.627981

**Published:** 2021-04-12

**Authors:** Daniel L. Drane, Nigel P. Pedersen, David S. Sabsevitz, Cady Block, Adam S. Dickey, Abdulrahman Alwaki, Ammar Kheder

**Affiliations:** ^1^Department of Neurology, Emory University School of Medicine, Atlanta, GA, United States; ^2^Emory Epilepsy Center, Atlanta, GA, United States; ^3^Department of Pediatrics, Emory University School of Medicine, Atlanta, GA, United States; ^4^Department of Neurology, University of Washington School of Medicine, Seattle, WA, United States; ^5^Department of Biomedical Engineering, Georgia Institute of Technology and Emory University, Atlanta, GA, United States; ^6^Department of Psychology and Psychiatry, Mayo Clinic, Jacksonville, FL, United States; ^7^Department of Neurological Surgery, Mayo Clinic, Jacksonville, FL, United States

**Keywords:** stimulation mapping, language, passive mapping, cerebral cortex, connectivity, socioemotional, memory, SEEG

## Abstract

Mapping of cortical functions is critical for the best clinical care of patients undergoing epilepsy and tumor surgery, but also to better understand human brain function and connectivity. The purpose of this review is to explore existing and potential means of mapping higher cortical functions, including stimulation mapping, passive mapping, and connectivity analyses. We examine the history of mapping, differences between subdural and stereoelectroencephalographic approaches, and some risks and safety aspects, before examining different types of functional mapping. Much of this review explores the prospects for new mapping approaches to better understand other components of language, memory, spatial skills, executive, and socio-emotional functions. We also touch on brain-machine interfaces, philosophical aspects of aligning tasks to brain circuits, and the study of consciousness. We end by discussing multi-modal testing and virtual reality approaches to mapping higher cortical functions.

## Introduction

Mapping of cortical functions in humans has provided substantial insights about the organization of the human forebrain. This review focuses on the mapping of higher cognitive functions, and will not address the mapping of primary sensory or primary motor cortices [recently covered elsewhere: (1)] nor the premotor or cingulate motor regions. We will describe the rationale for mapping in the context of its conceptual and historical development. We will then describe stimulation mapping and how it differs between subdural electrode stimulation and in the setting of stereoelectroenecephalography (SEEG). We will review language mapping and then describe broader applications of mapping to cognition and emotion and the potential for further research and technical development. Wherever relevant we will highlight controversies, key concepts, gaps in knowledge, and areas of present development.

## Rationale and Opportunity for Intracranial Monitoring for Understanding Seizure Networks and Conducting Neurocognitive Mapping

When seizures continue despite thoughtful medical management, epilepsy surgery is considered, often in turn resulting in invasive electrophysiology with subdural or depth electrodes. The ultimate goal of such studies is to test hypotheses about the network and location of seizure onset with a view to resective surgery, tissue ablation, or neuromodulation (2). In order to localize areas of hyperexcitability, reproduce seizures and aura, and map cortical function, electrical stimulation can also be performed while intracranial electrodes are in place, with this method arguably having afforded the greatest body of knowledge about cortical function through the last ~80 years. For much of this time in North America, this has involved a large craniotomy and placement of a subdural grid of electrodes on the surface of the brain, sometimes augmented with strip or depth electrodes. In contrast, in Europe, the approach to invasive studies has grown out of a more clinical approach with semiologic analysis, ultimately relying on less invasive depth electrodes with the overall method referred to as SEEG (3). While both methods started out as acute approaches, limited to the operating room, both rapidly developed into continuous extra-operative recording.

SEEG has been used more extensively in Europe for many years (4), but has recently become popular in North America, particularly since the use of minimally invasive procedures has increased (5). These have included the use of laser interstitial thermal therapy [LITT (6)], focused ultrasound (7), and neuromodulatory procedures [e.g., vagal nerve stimulation (VNS), responsive neurostimulation (RNS), and deep brain stimulation (DBS) of the anterior nucleus of the thalamus [DSBS-ANT (8, 9)]. With these less invasive surgical options available, patients and neurosurgeons are reluctant to use a larger procedure for evaluative purposes if the treatment is going to ultimately be more restricted and less invasive in scope. In this manuscript, we will briefly review the history of invasive EEG techniques and explore both the strengths and weaknesses of these procedures. We will discuss the use of such techniques to better understand seizure networks and brain connectivity and to study cognitive and emotional processes, then propose a research agenda that improves clinical practice and furthers our understanding of neural circuitry. The latter goal could include the standardization and dissemination of assessment techniques and the development of new cognitive and emotional testing paradigms that build upon current brain-behavior theoretical models and make use of modern technologies (e.g., augmented and virtual reality, videography, eye-tracking).

## History of SEEG and Neuropsychological Mapping

### Historical Antecedents of SEEG

In the late eighteenth and early nineteenth century, studies of electricity and its relationship to biology were in their infancy, with debate between Volta and Galvani about intrinsic vs. extrinsic electricity and electrical stimulation of movement (10). This debate was largely synthesized and resolved by Humboldt as Galvani's nephew first stimulated a freshly executed human cadaver in 1802, giving rise to bodily movements, inspiring the public imagination and likely contributing to the creation of the Frankenstein story. Crude localization of function was best inspired by Thomas Willis, but more fine grained cerebral localization principally occurred in the nineteenth century with clinical-pathological correlations and early motor mapping by Fritsch and Hitzig in dogs (11), and a more precise topography described by Ferrier in non-human primates (12–14). These works culminated in the “The Functions of the Brain” in 1876 (15), with later correlations to some clinical observations by Hughlings Jackson (16).

In the clinical realm, this new appreciation of the organization of the cerebral cortex developed alongside the other technical and research advances, such as stereotaxis and the string galvanometer for measuring electrical potentials. Most notably, shortly after David Ferrier's student Robert Caton recorded human brain potentials for the first time (17), Victor Horsley electrically stimulated an encephalocele then, at the behest of Hughlings Jackson, performed the first electrocorticography for epilepsy surgery (18). In 1909, Harvey Cushing reported the use of cortical stimulation in two cases of focal epilepsy (19, 20), with Emil Theodor Krause, in the same year, publishing the first map of the human motor cortex (21). This was followed by a more extensive map by Ottfreid Foerster—a major influence on Penfield's work and his approach to epilepsy surgery. In fact, the literal translation of Foerster's ideas into English and North America was so verbatim that we still use the German alternating current line frequency of 50 Hz in mapping studies till the present day—the original stimulators being simple step-down alternating current transformers.

While cerebral localization had gotten off to a somewhat pseudoscientific start, complete with racialized tropes, the phrenology of Gall and Spurzheim gave way to scientific cortical localization (22), as described above with support from the developing field of neuroanatomy, as well as to anti-localizationalist works in the early twentieth century (e.g., Karl Lashley). In clinical spheres, localization became the hallmark and unique method of neurology, growing from the prescient conjecture of Willis to an eventual clinico-pathological method of Paul Broca and, most productively, Jean Marie Charcot who essentially described the modern screening neurological examination (23). While localization in epilepsy reached a new summit in the works of Hughlings Jackson, epilepsy in the English speaking world did not keep up with the sophistication of the French fusion of Charcot's anatomical and clinical thinking that was directly mapped to the brain and its networks by Jean Talairach, the psychiatrist who pioneered, along with his collaborators in the 1960s, a new stereotaxic method and means of determining anatomical correlations to a standard atlas in functional neurosurgery [e.g., (24)]. While a fruitful research stereotaxic apparatus was invented by the surgeon Victor Horlsey and Robert Henry Clarke, ushering in a new era of neuroscientific discoveries, it was not used in humans until the late 1940s by the founding epileptologist Frederic Gibbs at Harvard, along with Robert Hayne, and was not accompanied by an overall anatomical and localizationalist approach to epilepsy (25). Instead, Talairach, working closely with the neurophysiologist and neurologist Jean Bancaud, developed the means to combine astute localization of seizure semiology, individualized anatomy with functional correlations, and a stereotaxic approach to hypothesis testing, culminating in the 1950s and 1960s into the method that we now know as stereoelectroencephalography (26).

While amplifiers and recording methods, along with the SEEG method were developing, it is important to note that electrical stimulation actually preceded multichannel electrophysiologic recording. Electrodes were thus crafted with stimulation in mind and this was an integral part of both intra- and extra-operative use of both subdural and SEEG-based methods. While stimulation subserves several functions, as mentioned above, we focus in this review on the application of stimulation for electrical stimulation mapping of function (ESM) which can overlap with elicitation of the seizure aura, passive mapping, and connectivity mapping.

### Historical Course of Cortical Stimulation Mapping

ESM involves the application of electrical current, typically to the cerebral cortex, in an effort to determine the potential contribution of a given region to a specific cortical function (e.g., sensory, motor, cognitive, linguistic, socio-emotional). While there are a few clear objectives, mapping of function is not always separate from stimulation to elicit after discharges and seizures. For example, stimulation during language testing may be found to reproduce ictal aphasia when a run of after-discharges or focal seizure is elicited in language networks. Similarly, mapping of function may help understand both which cortex is “eloquent,” but also elicit the seizure aura. Eloquence is a problematic concept and essentially equates to an observable function that is considered of high importance. However, under the right conditions and with appropriate testing, much, if not in theory all, of the cerebral cortex can be demonstrated to have a function–this is a major motivation for expanding the paradigms available for cognitive mapping. Nonetheless, eloquent cortex is conventionally considered to subserve key functions such as motor control and core components of language and speech. ESM has most frequently been employed intra- or extra-operatively in the setting of epilepsy and tumor surgery [(26–29)].

#### Drivers in North America

**Figure 1:**
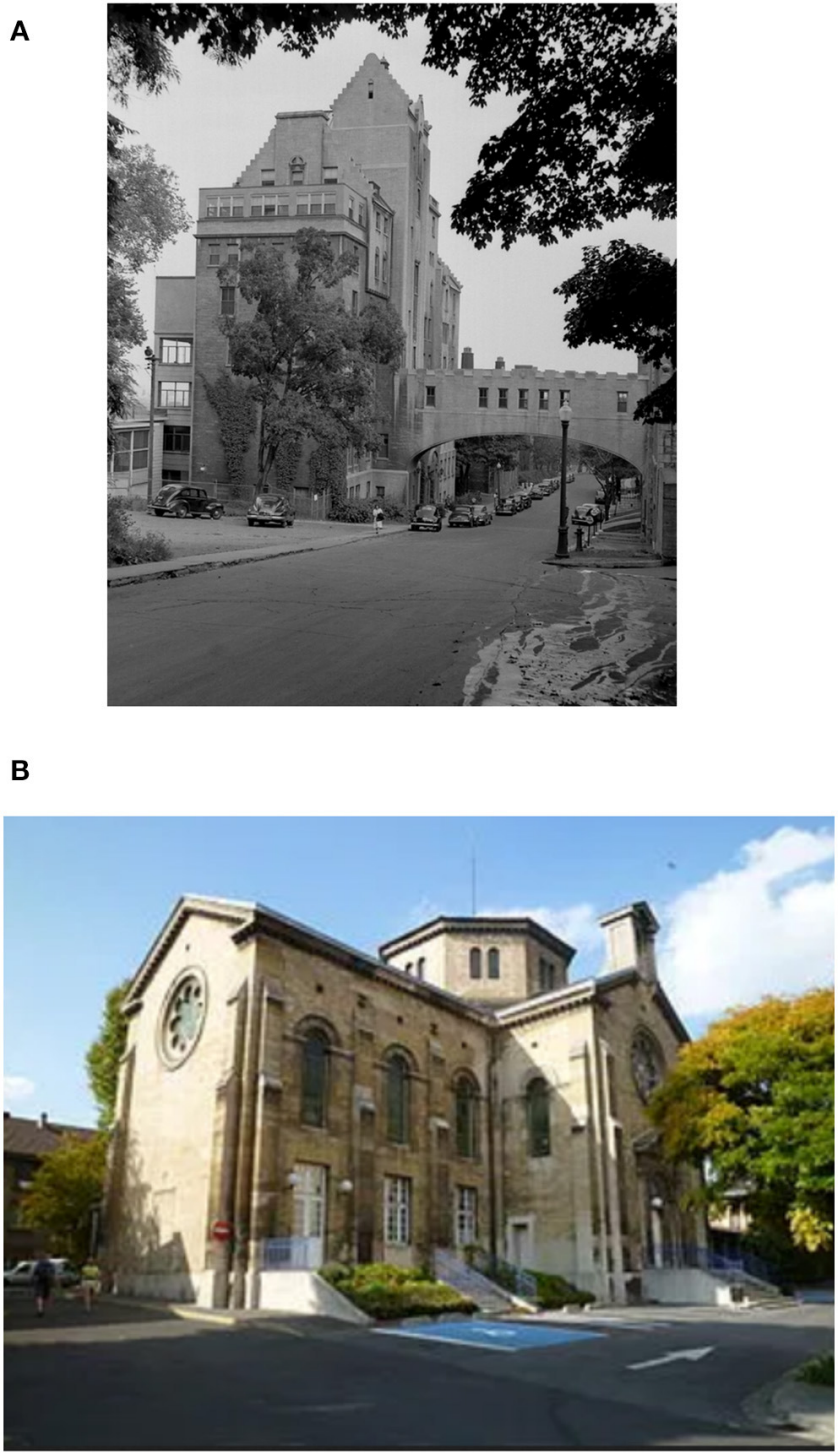
Historical sites central to the development of cognitive mapping: **(A)** the Montreal Neurological Institute, Montreal, Canada and **(B)** St. Anne Hospital, Paris, France.

Wilder Penfield, after studying with Foerster, went on to employ direct electrical stimulation to explore the sensorimotor cortex, experiential phenomena and language functioning (30–32). Prior to that time, in clinical practice, neurosurgeons were reluctant to operate on the language dominant cerebral hemisphere for fear of creating a language disturbance (aphasia), with the exception of procedures involving the occipital lobe or anterior frontal lobe. Therefore, the introduction of ESM for language mapping opened new surgical opportunities for many patients who would have been considered at risk of harm from an open resection procedure. Penfield, along with his colleague Herbert Jasper, created what they termed the “Montreal procedure” during the 1930s, which involved testing the surgical patient while undergoing ESM in an awake state (Penfield and Jasper, 195) (33) (See Figure 1). Their pioneering work led to many discoveries and advancements in our knowledge of neural networks underlying language, and specifically demonstrating that such networks were much more complex and extensive than the classic language network postulated by Broca, Wernicke, Lichtheim, and others (34–37). They also showed that they could elicit both the auras of patients, which can be useful for localizing seizure networks, as well as experiential phenomena such as rich memories of events that included sensory phenomena (e.g., “I am in my grandma's house and I can smell the cookies baking”). With the advent of current regulated bipolar stimulation devices, as well as more adherence to safety limits, less electrical current is applied directly to the cortex, and rarely, if ever, are such contextually rich phenomena seen with cortical surface stimulation.

George Ojemann, one of the founding members of the regional epilepsy center at the University of Washington (UW), built upon these ESM experiences, devoting much of his research career to the use of mapping paradigms to better understand cortical function. The UW program was the second regional epilepsy center in the US, following the University of Virginia, with programs funded by a project grant from the National Institutes of Health (NIH). His seminal work in 1989 (38), which included careful language maps of 117 presurgical temporal lobe epilepsy (TLE) patients, clearly demonstrated that critical language sites are much more broadly represented across the cortex than had been conceived. Investigators heavily relied upon visual (picture) naming approaches, which were likely tapping into the widely distributed networks required for one of the more complex actions carried out by humans (i.e., to name an object requires a large swathe of cortex from the primary visual area, unimodal and polymodal association cortices including lexical access, speech monitoring, and production) [see (34, 36, 39)]. There are actually multiple stages to this process, which can be carried out for some stimuli under 1 s, while more complex material may require slightly longer (e.g., 5–8 s for some complex stimuli). The Ojemann work, which included the projects of many trainees and collaborators, led to greater knowledge of reorganization of language and normal atypical variants (e.g., extent, spatial reorganization, contributing factors) and increased knowledge of primary vs. secondary languages and their neural distribution (e.g., primary languages appear to be more focally distributed than secondary languages) (40–45). This work also delved into the study of memory and led to some provocative findings which are being given more credence with the study of focal lesions resulting from our current epoch involving the introduction of minimally invasive surgical techniques. For example, ESM memory paradigms suggested that disruption of lateral cortex could disrupt episodic memory (46, 47), and it has recently been borne out that focal lesions in such subregions can cause lasting damage to memory even when medial TL structures are spared (48). The extent to which such functions could be localized to these lateral regions remains to be determined, and it may be that both medial and lateral aspects of the TL are simply contributing TL larger, interactive networks.

Investigators at the Cleveland Clinic have also made some important contributions to our knowledge of language networks using ESM. In particular, Lüders and colleagues discovered that application of electrical stimulation of the basal temporal lobe region (fusiform gyrus) of the dominant hemisphere at a high intensity produced a significant aphasia in a subset of patients involving disruption of both comprehension and speech output (49–51). This finding sometimes seems underappreciated by the neurological community, and this region is still not routinely assessed in many clinical investigations. Of note, lower intensity stimulation of this region only resulted in naming deficits. This area was dubbed the basal temporal language area.

While Lüders and colleagues initially reported no significant language deficits resulting from resection of this region (51), subsequent studies have indeed reported impairment (52–54). The original work of Penfield also indicated that resection of this region could result in significant confrontation naming problems (32). As electrical stimulation led to comprehension deficits as well as other language symptoms, this may be additional data suggesting a more posterior location of a region that is important for single word comprehension. One group has published a couple of case studies using subdural grid mapping of epilepsy patients which suggest that the posterior basal temporal language area may be most important for relating visual images to phonological content (55). Building upon these findings, a more recent electrophysiological study, which employed cortico-cortical evoked potentials to explore regional connectivity, revealed connectivity between the basal temporal language area and a posterior language area (56). It is also possible that this reflects disruption of the ventral auditory processing pathway, perhaps when including basal temporal white matter.

#### Benefits and Limitations of ESM

The most obvious benefit of ESM is its potential to spare eloquent functions, which typically include language and sensorimotor function, but really should be considered more broadly to include polymodal, cognitive and emotional processing components. Limitations of ESM primarily involve limits related to the coverage that is possible through grid mapping (29) and the sparse sampling of SEEG. In general, systematic coverage is provided for a limited range of the cerebral cortex. Another key limitation is that not only is the node being stimulated affected, but it seems likely, given conveyance of evoked potentials to other regions during stimulation, that what is observed clinically could be a network effect. Other limitations include a lack of cross-center standardization, and a lack of validated, standardized tasks to cover most cognitive abilities.

### Historical Course of Mapping With SEEG

#### European Contributions

As described above, Jean Talairach designed a robust system which allowed patient-specific mapping of cerebral anatomy to a coordinate space that included functional correlations (4). This impressive registration before the computed tomography and magnetic resonance imaging eras used ventricular position and size derived from pneumoencephalography and angiography to delimit major vessels, particularly arteries, as well as the anterior and posterior commissures. At the same time, Jean Bancaud finished his higher doctoral thesis on the correlation between neuropsychological deficits and EEG in patients with brain tumors. He saw the value of using Talairach space in defining three-dimensional representations of seizures and their propagation, and a new method was born. With the advances in technology and regulations, it became possible to move from intraoperative “acute” to intraoperative prolonged and even ambulatory “chronic” SEEG recordings in France [see (57) for a brief history of epilepsy surgery in France].

While Penfield and Jasper primarily relied on interictal spikes and cortical stimulation for his resections, Talairach and Bancaud were able to obtain ictal recordings, sometimes elicited by pro-convulsant drugs such as pentylenetetrazol, and replicated as components or as a complete sequence to understand the spatiotemporal evolution of spontaneous seizures. While paroxysmal evoked responses were also obtained by both single shock and train stimulation (4, 58, 59), this approach, in contrast to that of Penfield and Jasper, relied on the careful analysis of seizures, rather than resection based on interictal abnormalities.

There were several differences between North American and European approaches to stimulation and mapping. While North American approaches typically demarcated functional mapping from analysis of the patient's epileptic network, this was often not the case in Europe. For example, examination might occur when eliciting the seizure or components thereof, as described above. While 50 Hz stimulation was prominent in North America, the SEEG approach tended to emphasize the importance of tailoring stimulation frequency, amplitude, and train duration based on the location stimulated [e.g., (59)]. From a practical standpoint, low frequency stimulation is used for functional mapping of primary areas, for the study of functional connectivity, and of areas with a lower threshold for after-discharge and seizure (e.g., amygdala and hippocampus); high frequency stimulation, such as 50 Hz, is used for functional mapping of non-primary neocortical areas, and for triggering seizures. SEEG can be utilized to investigate and identify language networks in patients with epilepsy (60). In North America, there was a concern for “clearing” cortex that may be “eloquent” for resection, while the SEEG method tended to precede from the patient's semiology and examine function in relation to seizures. Later academic work in both arenas focused more formally on examining specific cortical functions—work that is far from exhausted today.

While SEEG can be used, unlike cortical surface mapping, to map the functions and connections of fiber bundles [e.g., (61)], much of the work of electrical mapping of the language functions of white matter has been performed intraoperatively, particularly by Duffau and colleagues. This has been achieved by mapping during tumor surgery, involving stimulation of the cortical surface and white matter tracts as they move through the surgical zone in the setting of tumor resection (62, 63). They have combined this approach in an interesting fashion with the use of neuroimaging techniques (e.g., fMRI, DTI) and neuro-dissection techniques in a multimodal manner that has contributed to more localized analyses of function and the inclusion of pathways/connections to the sometimes exclusive focus of past researchers on gray matter/cortex only (64–67). Of note, however, one criticism has been that some of this work has been based on a more crude localization using photographs of the area being stimulated, which are then matched to the available preoperative MRI scans. Therefore, there is likely much more work that can be accomplished in this area as well. These investigators have demonstrated that naming dysfunction differs by the white matter pathway that is stimulated [e.g., semantic errors tend to occur during picture naming when stimulating the inferior frontal occipital fasciculus (IFOF) while phonemic paraphasia results from stimulation of the arcuate fasciculus (67)], and that disturbance of recognition of faces and objects can occur with stimulation of the non-dominant ventral visual processing stream (68, 69). They have also published data showing that seemingly small declines in language processes (including simple speech response speed on naming) can lead to significant decline in functional status (e.g., failure to return to work relates to slowed response rate on these tasks) (70).

#### Benefits and Limitations of Mapping With SEEG

While the SEEG method itself affords more access to anatomically distant but functionally related areas, with superior access to sulci and deeper cortical and subcortical structures than ESM, the trade-off involves a reliance on a sparser sampling of the cortical regions of interest. A related benefit is that when a patient is implanted thoughtfully, large functional networks can be probed, rather than adjacent fragments of multiple networks as is typically the case with a subdural approach. While use of a 3D-grid approach has been described to overcome sparse sampling (71), this is largely at odds with the overall localizationalist and network thinking of the SEEG method and is generally not recommended—the large number of electrodes in one region, at the expense of covering other hypotheses or parts of the network, may increase hemorrhage risk, although electrode density as a specific risk for hemorrhage has not been studied. Furthermore, skull thickness can be a limiting factor in sampling certain areas (such as anterior temporal region). This is a major consideration in younger children. Finally, SEEG is less invasive than ESM using grids and strips, and this fits very well with the continued trend toward employing minimally invasive surgical procedures.

There are several limitations to mapping with SEEG. A major present obstacle is the learning curve in practicing the SEEG method. There is an uneven level of experience in SEEG mapping within centers which have used SEEG for decades and have well-established methods in place when compared to centers that adopted this method recently. The publication of the French guidelines on SEEG in 2018 is an effort to standardize the practice across centers and offer recommendations for those who are implementing the method (72). The interpretation of positive or negative responses during stimulation can be challenging from a functional standpoint. One of the key concepts that can impact functional mapping in SEEG is charge density given the relatively small surface area stimulated. Theoretically, the stimulation effect is local, however, a distant effect of stimulation is also likely and should be considered when interpreting stimulation results, particularly given that intercontact spacing typically means that white matter is also stimulated [human cortical thickness varies from about 1–4.5 mm, e.g., (73), and 2 mm contacts are typically not <1.5 mm apart, often more] (74, 75). Certain charge densities and frequencies may have an inhibitory effect on certain cortical areas, or may result in inhibition of downstream targets (e.g., with stimulation of the prefrontal cortex), positive clinical phenomenon, or inactivation of a network that extends beyond the area stimulated (e.g., some aphasic effects in language mapping). For all of these reasons, mapping a complex function such as language requires experience and analysis with subdural electrodes and perhaps still more with SEEG, whereas mapping primary motor or sensory function is usually straightforward despite a lower spatial resolution. Therefore, mapping must be used thoughtfully and is best tailored to individual patients and functions. Probing the presence and the effect of a focus in a large interconnected network can be accomplished with SEEG.

## Current Status of Cognitive and Emotional Mapping with SEEG

### Stimulation Parameters

The parameter space for stimulation can be large, but fundamental neurophysiology significantly reduces this multivariate parameter space. The variables of stimulation are intensity, montage (e.g., bipolar vs. monopolar), duration, waveform, charge balancing, frequency, and train characteristics. While not considered in this review, issues of geometry, contact size and shape, and material are also important considerations. This parameter space is made more tractable by the safety limit in charge per phase with SEEG electrodes that limits amplitude and pulse duration (with 30 μC/cm^2^/phase based on animal data, electrode metal characteristics and pathological examining of human post-mortem tissue after chronic basal ganglia stimulation) (76). Waveforms have been explored to a greater extent in basal ganglia stimulation and typically a symmetrical charge balanced square wave is used in SEEG, despite other waveforms possibly providing more targeted stimulation at least in microstimulation (77)—this is yet to be studied in detail. Montage is determined by the volume of tissue that one desires to activate and there is a significant body of work pertaining to stimulation frequency for the cerebral cortex in mapping, as mentioned in a prior section [e.g., (26, 78)]. Subdural grid stimulation parameters are similar, except that a higher safety limit is typically accepted (~50 μC/cm^2^/phase), perhaps given short-circuiting over the pia and through cerebrospinal fluid, based on elemental pathologic analysis of resected temporal cortical tissue after acute stimulation and subsequent temporal lobectomy (79). While these factors all serve to make the stimulation parameter space quite tractable, this should not be confused with therapeutic stimulation parameters, or higher frequency stimulation that may be inhibitory—the parameter space is under-determined, but has gradually become better explored (75).

### Risks of Depth Electrode Placement

While SEEG is substantially less morbid and better tolerated than craniotomy with subdural grid placement, injury must be considered. Broadly, it is worth considering three categories of injury: Major (e.g., neurologic deficit that does not entirely resolve), minor (resolving complications), and subtle. We define the latter here as long term effects of electrode insertion in the absence of any complication. Regarding major and minor complications, the most common is hemorrhage. The largest study of complications of SEEG placement reveals a per-patient risk of hemorrhage of about 19%, and ~0.2% risk per electrode for hemorrhage, being symptomatic at a rate of about 0.05% per electrode. In this study it is noted that there is a measurable increase in hemorrhage, on a per patient basis, when the number of electrodes exceeds about 13 (80). Similar rates of symptomatic hemorrhage per electrode (0.04%) were found with a larger SEEG case series focused on broader SEEG practice (81). Overall, this is favorable to the potential harm of subdural grid placement which has a higher incidence of infection, subdural hemorrhage and cerebral edema (82).

Subtle injuries are less clear, given that studies have not yet been designed to prospectively identify these effects. Two fairly recent studies raised questions about the possibility that cognitive function, particularly memory, might be adversely affected by bilateral depth electrodes placed orthogonally through the hippocampi (83, 84). Nevertheless, both studies were retrospective in nature and limited by methodological imprecisions that could not be overcome after the fact (e.g., group differences that could not be controlled for in a retrospective clinical study). In contrast, similar studies have not been suggestive of this decline, and this has included work completed using neurostimulation devices that have been placed longitudinally along the hippocampus in both occipital and temporal lobes. Evaluations of this area have been the subject of several recent commentaries, which go into a great deal of detail, and suggest the need for prospective studies to overcome these limitations (85–87).

### Promise of Cognitive Mapping With SEEG

#### Passive vs. Active Stimulation Mapping

Historically, as we have covered thus far in this manuscript, cognitive mapping for epilepsy presurgical candidates has involved administration of a cognitive paradigm while high frequency electrical stimulation is actively delivered. One drawback to this approach is that it requires the presence of an epileptologist to perform the stimulation and monitor the live recording for after-discharges and seizures, as well as a neuropsychologist or cognitively oriented neurologist. While 50 Hz stimulation is often appropriate for cognitive mapping, outside of the sensorimotor cortex and hippocampal formation, with both a broader range of brain structures accessed and with a greater sophistication in planning stimulation, other frequency, and train parameters can be, and perhaps ought to be chosen. With higher frequency stimulation, seizures are more likely, which abort the attempt to study cognition, or contributes to after discharges which can distort assessment results by disrupting broaders network regions in more formal cognitive assessment. This cannot be entirely avoided with altered stimulation parameters, but the occurrence of focal seizures can be of some assistance in understanding the function of components of the seizure network. Nonetheless, several studies demonstrate the problem of seizures disrupting cognitive mapping. For example, in a study of 122 pediatric patients who received 50 Hz electrical stimulation mapping, sizeable percentages of patients experienced after-discharges (77%) and seizures (35%) (88). In another sample of 57 adults undergoing active mapping, seizures occurred at a similar rate (33%); in a subset of this sample who underwent language assessment, 17% experienced seizures that disrupted mapping attempts (89).

**Figure 2:**
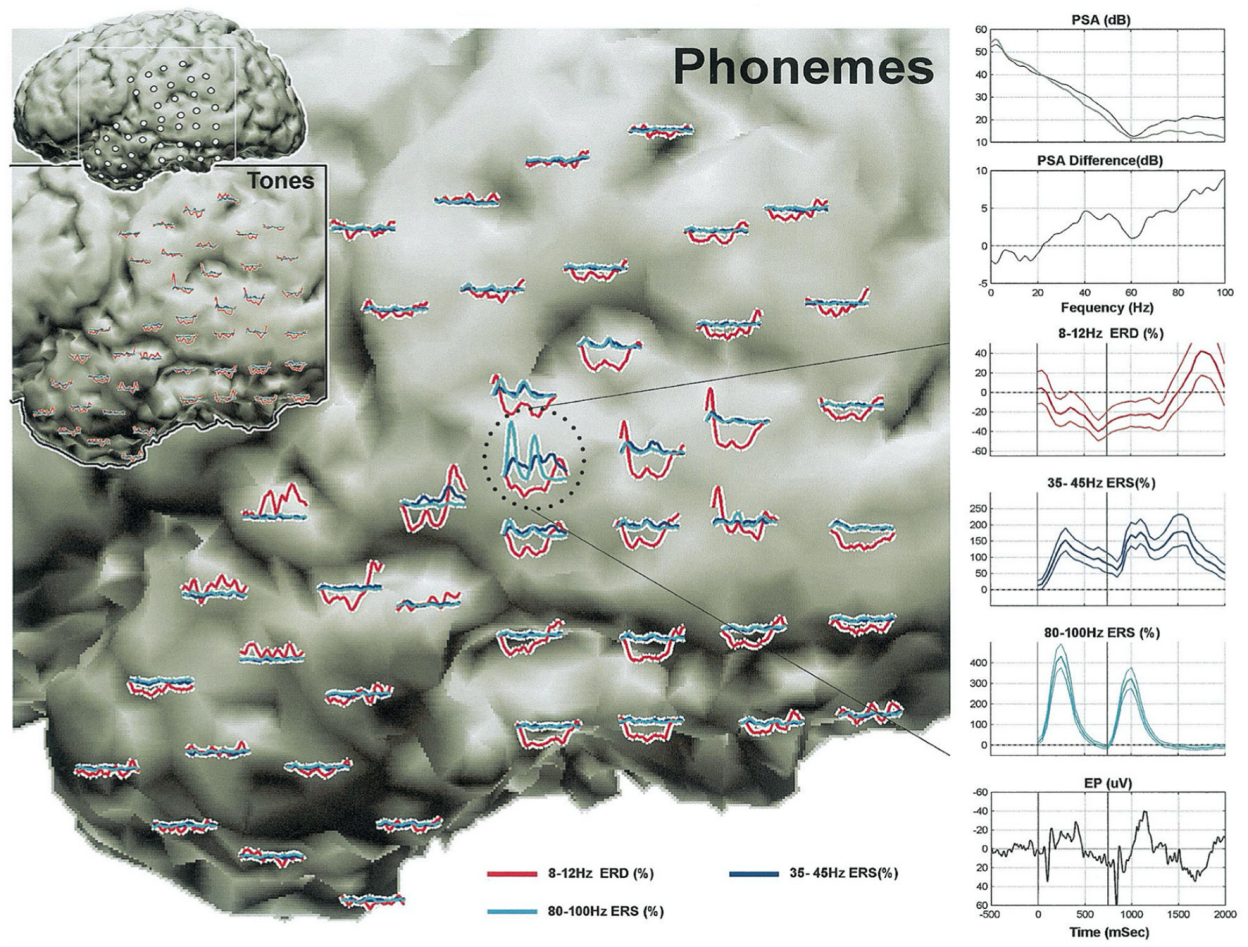
Cortical activity during a receptive language task. From the first study of passive mapping of electrocorticographic activity during a receptive task of distinguishing tones from phonemes by Crone et al. (90). Indices during perception of tones (lower left inset with black border) vs. phonemes (expanded view of left temporal lobe). Plots of event-related power augmentation/suppression are color-coded according to frequency, and correspond to the electrode locations depicted in the upper left corner inset (white frame indicates borders of the expanded views). Detailed plots in the right column are derived from an electrode over the left superior temporal gyrus (circled). PSA, power spectral array; ESD, event related desynchronization; ERS, event related synchronization; EP, evoked potential. From Crone et al. (90).

In more recent years, passive mapping of cognition has been explored (See Figure 2). This has been an important advance given that it is theoretically more localizing than stimulation, which can activate a network while exerting local influence, theoretically also providing cognitive evoked potentials or latency information. Conversely, brain regions related to other components of the task may be activated, arguing for a lower spatial specificity in passive mapping. Keeping both of these ideas in mind, it seems reasonable to consider this approach to mapping as complimentary, and potentially capable of acting as a second independent source of information when considering the functional organization of the cerebral cortex. From a practical standpoint, passive mapping is performed by conducting behavioral paradigms during concurrent electrocorticographic recording. In general, electrocorticographic analysis of broadband higher gamma frequencies (70–110 Hz) is typically carried out during administration of a specific cognitive or sensory task (e.g., confrontation naming, verbal fluency) (91–94). Recording of high gamma activity is best done with a sampling rate of ECOG data that is 2- to 3-times higher than the frequency of recorded activity to avoid aliasing of waveforms and a high frequency filter above 300 samples per second (S/s), fitting with most centers recording at 1 kS/s or more. After ECOG data is obtained, time-frequency analysis is performed using the bipolar montage when SEEG electrodes are used (95, 96). Bipolar montage analysis excludes common mode signals, such as muscle activity, whose high-frequency components can mimic high-gamma oscillations (97). Time-frequency analysis of event-related, high-gamma activity helps delineate the network involved in a given cognitive task (picture naming). For instance, high-gamma oscillations recorded at the onset of an auditory naming task is indicative of perceptual processing, while high-gamma activity at the end of the response represents motor processing.

Studies contrasting active and passive paradigms for language (particularly expressive) have shown reasonably equivalent results at least with respect to critical regions of common overlap [sensitivity (98, 99)]. Indeed, changes in gamma activity during passive language mapping do appear to overlap with language areas identified through electrical stimulation mapping (91, 99–102). In contrast, however, passive mapping paradigms find more regions related to task performance in general, as above, so these techniques do differ with regards to specificity, and this can lead to problems with determining core regions essential to a cognitive task. Although limited in scope, passive mapping appears efficacious when comparing mapping findings to postsurgical results; here, resection of regions that were associated with event-related gamma oscillations in passive mapping was associated with post-operative language deficits (103, 104). Much more work is required in this area, as larger studies are required that cover all behavioral paradigms of interest. Outcome validity studies are also woefully lacking for traditional active mapping paradigms as well.

Passive mapping may have some practical and accuracy advantages over active stimulation. First, passive mapping is more time efficient which lends itself well to children or adults who have difficulty completing more lengthy sessions of active cortical stimulation mapping or who cannot tolerate unpleasant sensations that can sometimes accompany stimulation mapping. It also avoids artificial perturbation of the brain, and thus reduces overall risk for after-discharges and seizures. Passive mapping is suggested to be more sensitive to localized cortical areas (e.g., as defined by language mapping: Broca's, Wernicke's, sensorimotor, basal temporal) than electrical stimulation mapping (102). In contrast, one potential limitation with passive mapping compared to active stimulation mapping, is that passive mapping, like fMRI activations, cannot determine whether regions are essential for a given cognitive task rather than simply being involved in the task (60, 105). If a region is not essential, it can still typically be included in a proposed surgical resection/ablation without harming overall function. Despite the limitations of stimulation mapping, and the potential to disrupt function more broadly than recognized or desired, it does create a “functional lesion” that may more accurately reflect the possible effect of surgical intervention. An interesting research proposition would be to use passive mapping to locate potential regions of eloquent brain tissue, and to follow this up with stimulation mapping of these areas in an attempt to better determine the potential effects of surgery.

#### Use of Machine Learning to Decode Passively Acquired Electrocorticography

**Figure 3:**
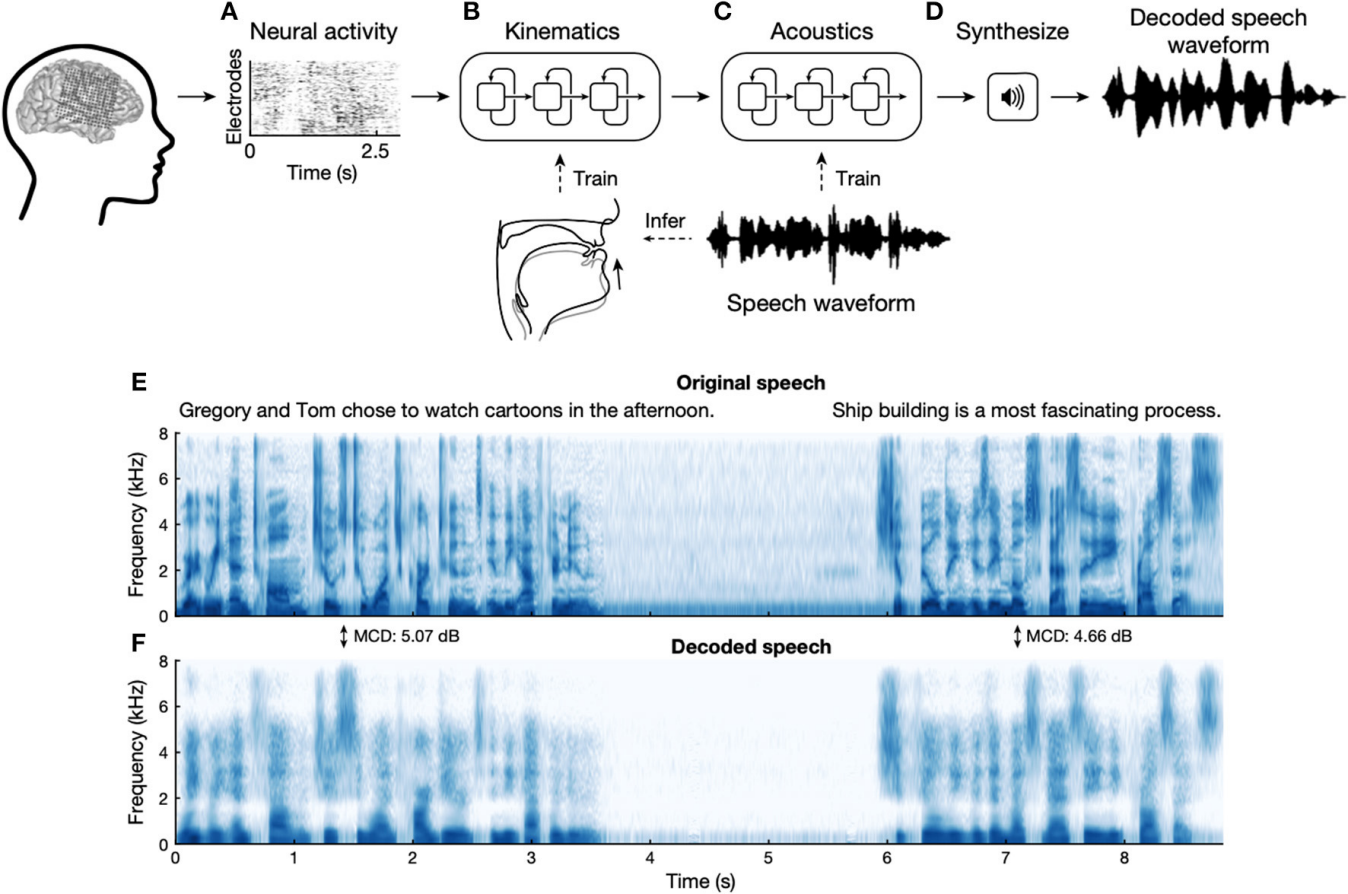
Speech synthesis from recorded electrocorticogram during spoken sentences. **(A)** The neural decoding process begins by extracting relevant signal features from high-density cortical activity. **(B)** A neural network decodes kinematic representations of articulation from ECoG signals. **(C)** An additional algorithm decodes acoustics from the previously decoded kinematics. Acoustics are spectral features extracted from the speech waveform. **(D)** Decoded signals are then synthesized into an acoustic waveform. **(E)** Spectrogram shows the frequency content of two sentences spoken by a participant. **(F)** Spectrogram of synthesized speech from brain signals recorded simultaneously with the speech in **(E)**. From Anumanchipalli et al. (106).

While brain-machine interfaces are generally beyond the scope of this review, there is topical overlap with passive cognitive mapping and research directed toward the decoding of speech. Given the likely problems of under-determining activity in a single brain region from sparse sampling with SEEG, this work has relied on grids, including higher density research grids. While this represents a large body of work that shares some similarities with work on brain-machine interfaces, the most notable current achievement is the transcription of speech while recording from language-dominant frontal opercular region (106), that performs quite well with simple sentences (See Figure 3). This decoding of neural activity in relation to behavior is particularly suited to machine and deep learning, where a classifier can be built or trained based on neural activity and an objective measure of behavior. These approaches will provide practical and theoretical insights into the organization of cortical function, as well as potentially providing a wide-array of brain-machine interfaces.

#### Connectivity, Cognition, and Philosophy

While some notions of localization focus on a one to one mapping of function and brain tissue, it is obvious that functional territories cannot act in isolation. A less extreme form of localizationist thinking might hold that cortical tissue maps well to function, with it taken as implicit that subcortical structures are necessary for input and output. When considering behavior, obviously large networks, typically with multiple cortical waypoints are involved. When considering the abstractions of cognitive psychology, there can be mapping of abstract functions to some brain regions, but the agreement of neurological thinking and the functionalist concepts of cognitive psychology are imperfect (take episodic and semantic memory for examples, where these concepts do not map directly to a brain region or perhaps even a circuit). Overall, this mismatch results in a philosophical approach that grew out of medical materialism, by way of Sellars (107) and Feyerarbend (108), that has come to be known as *eliminative materialism* (109) where *folk psychological* abstractions are eliminated in favor of an ontology based around neural mechanisms [see (110), for discussion]. This is critical to mention for three reasons: Firstly, we should keep this in mind when developing new tasks that aim to test particular brain networks. Secondly, this is likely a fruitful general line of research in neuroscience, behavior, and cognitive psychology. Lastly, it marries with a vastly increased interest in neural mechanisms in philosophy and particularly in the philosophy of mind. This latter scenario is beginning to provide productive common ground for more sophisticated understandings of consciousness, cognition and brain function.

#### Connectivity: DTI, fMRI, CCEPs, and Limitations

As we have thus far argued, an appreciation of connectivity is critical for understanding the correspondence between cortical functions and anatomical networks. There are several ways to determine and define connectivity. It should firstly be emphasized that the gold-standard of brain connectivity remains neuroanatomical tracing. These methods are based around the transport of stereotactically injected dyes, radiolabels or detectable proteins into the brain so that neurons projecting into the area of injection, or axons projecting from this region, can be visualized, often by autoradiography or histological methods and microscopy. Neurotropic viruses such as strains of herpes and rabies have also been used for tracing, which can be polysynaptic. More recently, viral vectors have been the principle means of determining afferent and efferent connections of a given brain region, and by using genetic methods, even of a particular cell type [see (111, 112)]. Needless to say, these methods cannot be performed in humans, meaning that our most detailed knowledge of veridical connectivity comes from non-human primates and inferences from other species. This leaves us with inference and indirect methods to determine connectivity and cortical networks essential for cognition in humans, one of which is based on and related to electrical stimulation mapping.

**Figure 4:**
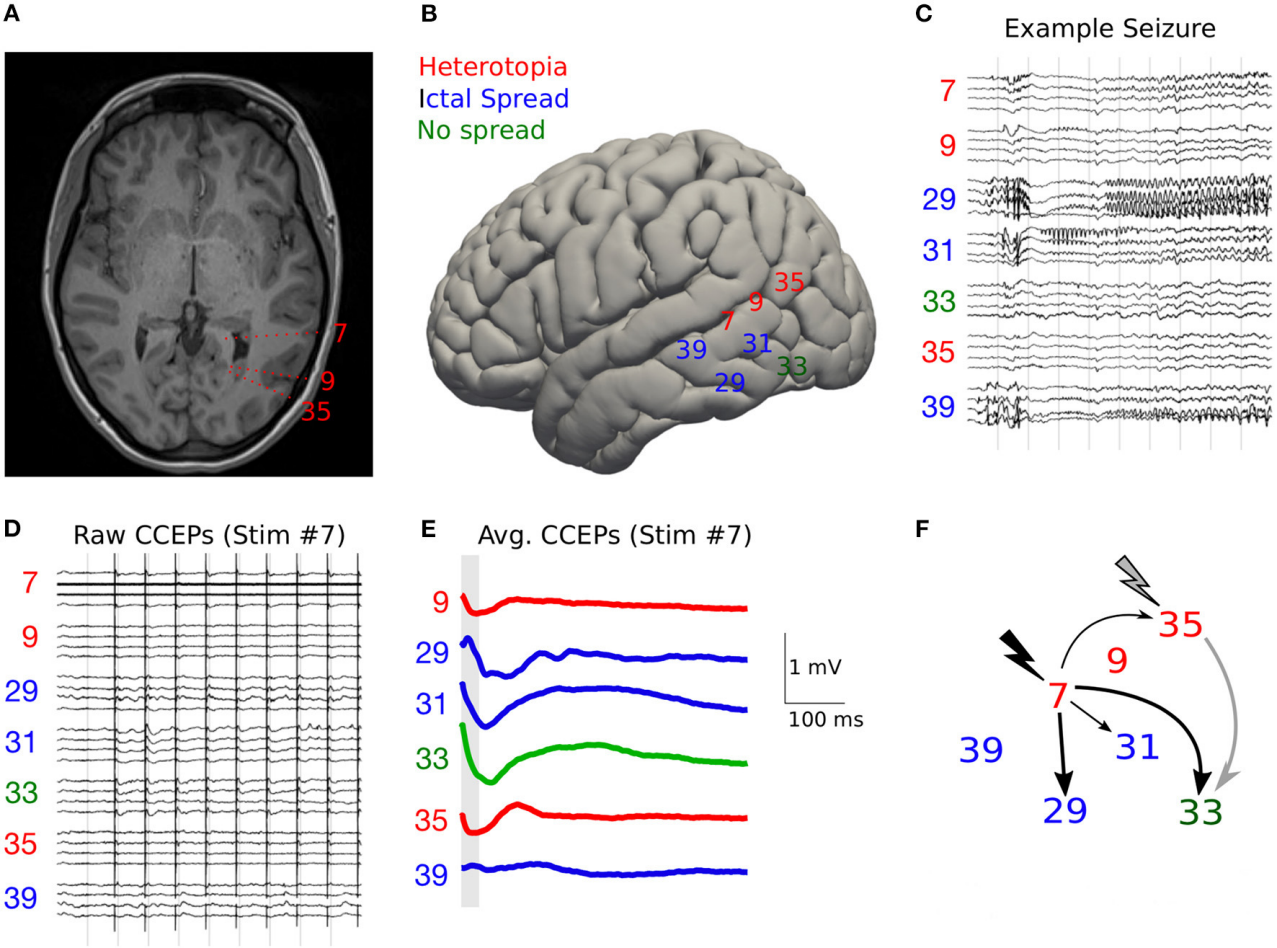
Example of using CCEPs to study effective connectivity. **(A)** Axial MRI Brian (T1) showing two periventricular nodular heterotopias in the tirgone of the left lateral ventricle and the trajectories of electrodes 7, 9, and 35, with dashes showing the approximate location of the 10 contacts of each recording electrode. **(B)** The approximate lateral entry points of pertinent left-sided SEEG electrodes are shown as electrode numbers. **(C)** An example spontaneous left sided seizure onset is shown with gamma activity on electrode 29 contacts 1–3 (posterior hippocampus). **(D)** Raw cortico-cortical evoked potentials (CCEPS) triggered by 1 Hz bipolar stimulation anterior heterotopion (electrode 7 contacts 3–4). Evoked potentials with peak to trough amplitude >250 μV are evident on electrodes 29, 31, and 33. **(E)** Averaged CCEPs with 20–50 ms (gray bar) window of interest shown. Only the largest amplitude (root mean squared amplitude (RMSA) CCEP (taken over all 10 contacts) is shown for each electrode. **(F)** Connectivity map for stimulation to electrode 7 (black) and 35 (gray). Thick arrows represent CCEPs with RMSA >200 mV, thin arrows represent CCEPs with RMSA >100 mV. From Dickey et al. (116).

From the human neuroimaging literature, the indirect methods of determining human “connectivity” is often divided into *structural, functional*, and *effective* connectivity (113). *Structural* connectivity, not to be confused with neuroanatomical tracing, typically refers to diffusion imaging-based methods such as probabilistic and deterministic tractography. There are numerous limitations to this method, when sharp axonal branches or curves cannot be followed, the direction is not determined, nor the length of a given set of axons be determined as identical to that of the larger bundle of fibers that can be detected [see (114)]. *Functional* connectivity need not represent anatomical connectivity at all: This is where a functional assay is used, and correlated activity is determined by blood oxygen level desaturation (as in functional MRI), or perhaps by intracranial EEG. The problem is that simultaneous activation can result from two structures having a shared input. For example, activation of subcortical nuclei can result in faster activity across the cortex, but it does meaningfully represent “connectivity” between these cortical regions. *Effective* connectivity is where a connection is implied if a recording site's activity is changed by perturbation of another site. This provides causal information, but typically cannot determine if connectivity is direct or indirect. An example, pertinent to the present review of functional networks is cortico-cortical evoked potentials [CCEPs (115)], which are perhaps the closest we can get to a measure of anatomical connectivity in the human cortex (See Figure 4). While this technique was first described by Brazier (117), by which time it may have already been appreciated by Bancaud and Talairach, it has undergone something of a recent revival. This technique is a boon for studies of human forebrain connectivity, especially as we attempt to divide the cerebral cortex into discrete functional networks. For example, this approach to using low-frequency stimulation, longer than the time of the complete evoked potential and typically 1 Hz, and its resultant CCEPs, has been used to examine connectivity between language areas (118, 119), within the motor cortices (120). This has been of assistance in mapping and in sparing white matter intraoperatively, to preserve connectivity between the anterior and posterior language areas. For example, in one patient a 50% drop in CCEPs amplitude was associated with a long-term language deficit (61).

## Functional Mapping of Cognition and Emotion—Anticipating Future Directions

### Cognitive Function

While much progress has been made in the mapping of cognitive function, there remains considerable variability in the techniques and paradigms used (29), there are many aspects of cognition that never get assessed, and the determinations of the full networks responsible for a function is often not determined. The focus of cognitive mapping has primarily been language, and to a lesser extent, aspects of memory. This is rightfully so, as the early days of epilepsy surgery were marred by poor outcomes of cases such as Henry Molaison (121), where the severity of the resulting amnestic state overshadowed any other aspect of the case. As we have gotten better at avoiding these catastrophic outcomes, and even lessening the poor cognitive outcomes through improved testing and minimally invasive surgical options, the focus of the field can now broaden to potential deficits which were previously underappreciated and potentially overshadowed. Initial findings suggest that some of these overlooked deficits can have some profound effects on patients (70, 122). Most cognitive mapping efforts have focused on language, in particular visual object naming. Even in this well-hewn area there is little consensus on training approaches as well as best practices for clinical and research efforts. Table 1 lists a number of cognitive and socio-emotional functions for which there is some evidence of structure-function knowledge derived primarily from cognitive mapping procedures.

**Table 1:** Chart of positive neural stimulation sites and specific neuropsychological functions (selected sample of representative studies).

**References**	**Function assessed**	**Region of stimulation**	
	**Language**	**Left hemisphere**	**Right hemisphere**
	**Visual naming**		
Ojemann et al. (38)	Visual naming (general)	Cortical stimulation across language dominant temporal lobe, frontal lobe, and parietal lobe sites (with significant variability across subjects)	N/A
Hamberger et al. (123)	Visual naming (objects)	Posterior temporal lobe regions	No effects of stimulation
Duffau et al. (124)	Visual naming (objects)	Dorsal PMC and underlying white matter	N/A
Ulvin et al. (125)	Visual naming (objects)	Stimulation of VTC led to naming deficits (particularly the FG and OTS)	Stimulation of VTC led to naming deficits in a single patient with right TL language, but no other subjects
Sarubbo et al. (126)	Visual naming (general)	STG, MFG, MFG WM, AG, AG WM, MTG, ITG, STG WM, IFG WM, SMG, insula, lateral FOC, ITG, WM, MTG WM, IFG, SMG WM, lateral FOC WM, FG	AG WM, AG, MTG, WM
	**Paraphasic errors during stimulation**	**Left hemisphere**	**Right hemisphere**
Leclercq et al. (67)	Phonemic paraphasic errors during visual naming	AF	No disruption with stimulation
Leclercq et al. (67)	Semantic paraphasic errors during visual naming	IFOF	No disruption with stimulation
Maldonado et al. (127)	Phonemic paraphasic errors during visual naming	PostAF (WM)	No disruption with stimulation
Miozzo et al. (128)	Semantic paraphasic errors during visual naming	Mid-middle temporal gyrus	No disruption with stimulation
Miozzo et al. (128)	Phonemic paraphasic errors during visual naming	Middle and posterior STG	No disruption with stimulation
	**Auditory naming (naming to description)**	**Left hemisphere**	**Right hemisphere**
Hamberger et al. (123)	Naming to verbal description (definitions) presented orally	Anterior temporal lobe	No disruption with stimulation
	**Transmodal naming**	**Left hemisphere**	**Right hemisphere**
Abel et al. (129)	Visual and Auditory Naming of Same Semantic Concept (e.g., famous person)	Anterior temporal lobe	No disruption with stimulation
	**Proper noun naming**	**Left hemisphere**	**Right hemisphere**
Abel et al. (129)	Famous person naming	Anterior temporal lobe/temporal pole	No disruption with stimulation
	**Language comprehension**	**Left hemisphere**	**Right hemisphere**
Sarubbo et al. (126)	Comprehension	SPL, STG, insula, SPL WM, SMG WM, MFG WM, STG WM	MFG WM, STG, hippocampus, MFG, STG WM, AG WM, MTG WM, AG, insula, ITG WM, ITG, PostCG, SMFG, MTG
	**Semantic processing**	**Left hemisphere**	**Right hemisphere**
Ulvin et al. (125)	Picture matching (semantically related)	No disruption from stimulation of VTC	No disruption from stimulation of VTC
Sarubbo et al. (126)	Semantic processing	MTG WM, insula, MTG, STG, hippocampus, MG WM, ITG, FG, ITG WM, STG WM, MFG, IFG, IFG WM, putamen, lateral FOC, FG WM, SFG, WM	No disruption
	**Reading**	**Left hemisphere**	**Right hemisphere**
Roux et al. (130)	Oral reading	Inferior aspect of pre- and Post CG, SMG, AG, and posterior STG, IFG, MFG, posterior MTG	Inferior aspect of pre- and Post CG, IFG
Roux et al. (130)	Articulation errors in oral reading	Inferior aspect of Pre- and Post CG	Inferior aspect of Pre- and Post CG
Roux et al. (130)	Ocular-induced reading errors	IFG	IFG
Sarubbo et al. (126)	Reading	ITG, FG, MTG, IOG, ITG WM, MTG WM, FG WM, IOG WM	ITG, ITG WM
Sabsevitz et al. (131)	Reading	Lateral fusiform gyrus (VWFA)	N/A
	**Acoustic responses/disruption**	**Left hemisphere**	**Right hemisphere**
Sarubbo et al. (126)	Acoustic responses	STG, STG WM, MTG	STG, MTG, STG WM, MTG WM
Sarubbo et al. (126)	Phonological	ITG, MTG, MTG WM, FG, IFG WM, STG, SPL WM, ITG WM, IFG, MFG, STG WM, AG WM, MFG WM, PostCG WM, SS, PreCG WM, AG, PostCG, SPL, SMG WM, PreCG, SMG, insula	No disruption
Duffau et al. (124)	Speech production	Ventral PMC and underlying WM	N/A
Sarubbo et al. (126)	Speech production	IFG, IFG WM, PreCG, PreCG WM, MFG, STG, insula, MFG WM, STG WM	PreCG WM, PreCG, MFG, IFG, insula, MFG WM, IFG WM, putamen
Sarubbo et al. (126)	Speech articulation	SMG WM, SMG, PostCG, PreCG WM, PostCG WM, PreCG, IFG, MFG, IFG WM, MFG WM, STG, AG WM, AG, insula, SFG WM	SMG, SMG WM, IFG, MFG SFG WM, MFG WM
	**Somatosensory**	**Left hemisphere**	**Right hemisphere**
Maldonado et al. (127)	Somatosensory	PostCG	PostCG
Sarubbo et al. (126)	Somatosensory	SPL WM, SPL, PostCG WM, precuneus, PostCG, PreCG, PreCG WM	SPL, PostCG WM, PostCG, SPL WM, SMG, AG, SMG, insula, pre-cuneus, STG, AG WM, PreCG
	**Motor function**	**Left hemisphere**	**Right hemisphere**
Blanke et al. (132)	Eye movements	Posterior portion of MFG, SFG; no response from IFG or precentral gyrus	Posterior portion of MFG, SFG; no response from IFG or precentral gyrus
Sarubbo et al. (126)	Eye movement control	MFG, MFG WM, SFG WM, PreCG, PreCG WM, SFG	MFG, MFG WM, SFG, SFG WM
Maldonado et al. (127)	Speech initiation/articulation	PO, horizontal portion of the lateral segment of the SLF III.	N/A
Sarubbo et al. (126)	Language initiation and motor planning	CN, SFG WM, SFG, MFG WM, insula, IFG, lateral FOC WM, IFG WM, MFG, putamen, lateral FOC	CN
Sarubbo et al. (126)	Motor	PreCG WM, SFG, PreCG, SFG WM, putamen, insula, MFG	preCG WM, SFG, putamen, SFG WM, PreCG, insular, MFG, MFG WM, PostCG, SLF, Post CG WM, IFG, IFG WM
Sarubbo et al. (126)	Motor control	SFG WM, SFG, MFG WM, MFG, CG, PostCG WM, PostCG, PreCG	SFG, SFG WM, MFG, CG, IFG, IFG WM, insula, MFG WM, putamen, precuneus
	**Consciousness/mental phenomenology**	**Left hemisphere**	**Right hemisphere**
Halgren et al. (133)	Déjà vu/“dreamy state”	Hippocampus and Amygdala	Hippocampus and Amygdala
Gloor (134)	Déjà vu	Lateral TL with spread to medial TL region	Lateral TL with spread to medial TL region
Bartolomei et al. (135)	Déjà vu	Entorhinal cortex, Perirhinal cortex hippocampus, amygdala (while this sensation could occur after stimulation of any of these structures it was much more common after entorhinal stimulation)	Entorhinal cortex, Perirhinal cortex hippocampus, amygdala (while this sensation could occur after stimulation of any of these structures it was much more common after entorhinal stimulation)
Bartolomei et al. (135)	Reminiscence of scenes	Perirhinal cortex	Perirhinal cortex
Sarubbo et al. (126)	“Mentalizing”	No disruption	MFG WM, IFG, MFG, SFG, IFG WM, SFG WM, CG, CN, insula
	**Emotional responses**	**Left hemisphere**	**Right hemisphere**
Lanteaume et al. (136)	Experience of negative emotions	Amygdala	Amygdala
Lanteaume et al. (136)	Experience of positive emotions	Amygdala	No effect elicited in right hemisphere
	**Visual processing**	**Left hemisphere**	**Right hemisphere**
Sarubbo et al. (126)	Visual	FG, IOG WM, FG WM, MOG WM, IOG	AG WM, AG, IOG, MTG WM, MOG WM, hippocampus, FG, IOG WM, SPL WM, SOG WM, MOG, MTG, ITG, FG WM, STG WM, SMG WM
	**Visuo-perceptual/visual-spatial**	**Left hemisphere**	**Right hemisphere**
Vignal et al. (137)	Facial hallucinations	No effect of stimulation	Ventrolateral prefrontal cortex
Barbeau et al. (138)	Famous face recognition	Passive mapping with intracerebral recordings demonstrates early involvement of the FG simultaneously with the IFG, then multiple regions of the ventral visual WM stream, and finally involvement of the hippocampus (much more pronounced in right hemisphere than left)	Passive mapping with intracerebral recordings demonstrates early involvement of the FG simultaneously with the IFG, then multiple regions of the ventral visual WM stream, and finally involvement of the hippocampus (much more pronounced in right hemisphere than left)
Fernandez Coello et al. (68)	Recognition of faces and select objects	N/A	Stimulation of ventral visual processing stream (IFOF and ILF)
Roux et al. (139)	Spatial neglect	N/A	Posterior part of the right STG and MTG, IPL, and inferior post CG and IFG. SLF II and SOFF
Bush et al. (140)	Spatial navigation	Increases in low and high frequency theta power are observed at the onset of movement in the hippocampus and lateral temporal lobe regions	Increases in low and high frequency theta power are observed at the onset of movement in the hippocampus and lateral temporal lobe regions
Maidenbaum et al. (141)	Spatial navigation	Entorhinal theta band activity is related to task performance	Entorhinal theta band activity is related to task performance
Sarubbo et al. (126)	Spatial perception	AG WM, AG	SMG, SMG WM, AG, AG WM, STG, SPL WM STG WM, MFG WM, PostCG WM, SPL, CG, MTG, PreCG WM, MFG
	**Arithmetic skills**	**Left hemisphere**	**Right hemisphere**
Duffau et al. (142)	Multiplication/subtraction	AG	N/A
Yu et al. (143)	Subtraction—but not multiplication disrupted at right hemisphere sites	N/A	IPL and AG
	**Memory functions**		
Haglund et al. (144) and Ojemann et al. (46)	Verbal episodic memory	Disrupted by stimulation of lateral TL cortex	No evidence of disruption from right TL stimulation
Coleshill et al. (145)	Verbal episodic memory	Disrupted by stimulation of amygdala and hippocampus	N/A
Ezzyat et al. (146)	Verbal episodic memory	Memory was enhanced at some frequencies by stimulation of lateral TL cortex in setting of SEEG	N/A
	**Executive functions**		
Bonini et al. (147)	Metacognitive evaluation of accuracy estimates	SMA	SMA
Puglisi et al. (148)	response inhibition	No disruption with stimulation	Non-dominant FL

*AF, arcuate fasciculus; AG, angular gyrus; CG, cingulate gyrus; FL, frontal lobe; FG, fusiform gyrus; FOC, fronts-orbital cortex; IFG, inferior frontal gyrus; IFOF, inferior frontal occipital fasciculus; ILF, inferior longitudinal fasciculus; IOG, inferior occipital gyrus; IPL, inferior parietal lobule; ITG, inferior temporal gyrus; MFG, middle frontal gyrus; MOG, middle occipital gyrus; MTGG, middle temporal gyrus; N/A, no assessment was completed; OTS, occipital temporal sulcus; PMC, pre-motor cortex; PO, parietal operculum; PostAF, posterior arcuate fasciculus; PostCG, postcentral gyrus; PreCG, precentral gyrus; SFG, superior frontal gyrus; SLF II/SLF III, superior longitudinal fasciculus; SMA, supplementary motor area; SMG, supra marginal gyrus; SOFF, superior occipital frontal fasciculus; SOG, superior occipital gyrus; SPL, superior parietal lobule; STG, superior temporal gyrus; TL, temporal lobe; VTC, ventral temporal cortex; VWFA, visual word form area; WM, white matter*.

### Language

Language mapping often involves administration of relatively simple tasks to test basic, automatic speech functions, such as counting or reciting overlearned phrases. Object naming is considered the “gold standard” for mapping language and allows for a broad sampling of the language network; however, more targeted, multitask testing may be needed to increase sensitivity as naming alone has been shown to miss 31% of temporoparietal and 43% of frontal language sites (149). It is widely accepted that language involves a more distributed network than Broca's and Wernicke's area and that anatomically dissociable regions exist that are specialized for specific linguistic subroutines which interactively support the construct of language (34, 39, 150, 151). Regions spanning the ventral temporal and occipital lobes and fusiform area appear to contribute heavily to recognition (primarily right hemisphere, but some left) of visual objects and faces, while coexisting areas on the left are important for naming (152–156). Naming itself is a complex construct, which can differ by modality of stimulus presentation [e.g., naming sounds vs. naming pictures (157)], object type [i.e., different object types map to different brain region (158, 159)], and level of classification [e.g., proper nouns have been more associated with the temporal pole while common nouns seem more broadly distributed (160)]. Naming can also vary by task parameters such as having a patient name objects/persons based on verbal descriptions (e.g., “the current President of the US”) rather than based on a sensory representation [e.g., naming a washing machine based on the sound it makes or its visual image (161)]. The latter tasks require the subject to determine the semantic content before applying the name, and are therefore slightly more complex. Orthographic or written letter content seems to be managed by a posterior temporal component of this stream [e.g., visual word form area (131, 162, 163)]. Aspects of the mid superior temporal gyrus and sulcus are dedicated to processing phonology or speech sounds, consistent with adjacency to unimodal auditory association cortex, while more posterior areas of the superior temporal gyrus and inferior parietal lobule involved in phonological access and retrieval, and areas in lateral middle and inferior temporal lobe, posterior inferior parietal lobe (angular gyrus) and dorsolateral frontal cortex that are involved in processing semantics [i.e., the meaning of pictures, words, phrases, etc. (29, 150, 164, 165)]. And this is of course all contingent on patient-specific factors like intelligence (44), gender (166), and handedness (167, 168) to name but a few.

**Figure 5:**
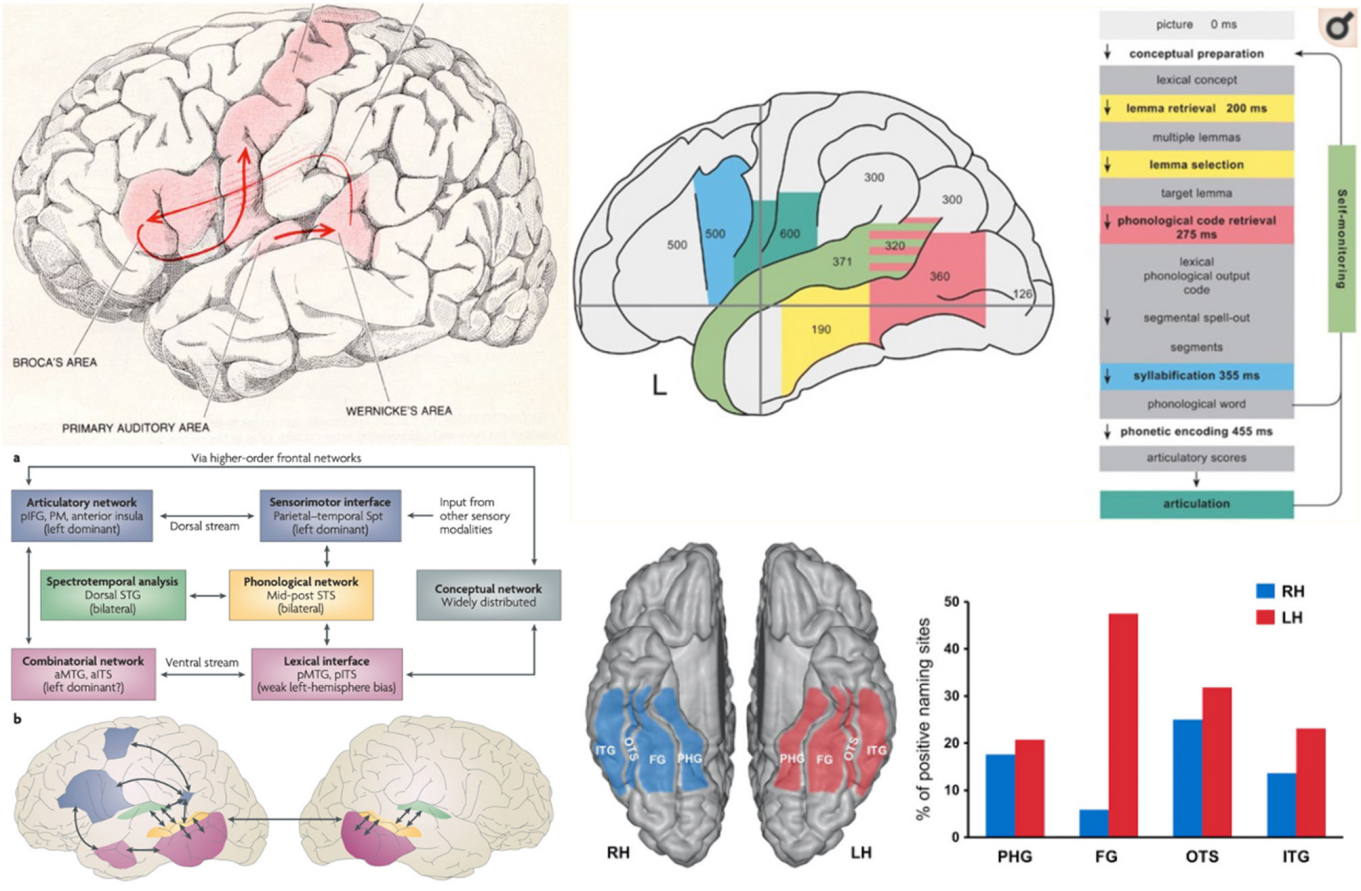
Models and Anatomy of Language Networks reveal the large area of the cerebral cortical involved in language. Top left: Geschwind's (169) illustration of the Broca-Wernicke model of language. Top right: Indefrey's (170) model of cortical activity (showing evoked potential latencies by region in milliseconds) during a confrontation naming task, demonstrating some of the cortical extent of language processing. Bottom left: Hickok and Poeppel (39) dual stream model of the cortical anatomy of auditory language comprehension and response where auditory processing is bilateral and involves bilateral superior temporal sulci and unimodal auditory cortex from which activity is conveyed to either a dorsal stream for, motor and articulatory analysis and phonological representation, vs. a ventral stream for lexical and conceptual representation (aITS, anterior inferior temporal sulcus; aMTG, anterior middle temporal gyrus; pIFG, posterior inferior frontal gyrus; PM, premotor cortex). Bottom right: From Ulvin et al. (125), showing the regions in which stimulation can result in specific naming deficits (“positive naming sites”), identifying the crucial role of the dominant fusiform basal temporal region in naming (ITG, inferior temporal gyrus; FG, fusiform gyrus; OTS, occipitotemporal sulcus; PHG, parahippocampal gyrus).

Given the complexity and multidimensional nature of language, there is a need to develop and use tasks that differentially engage or drive these linguistic processes so mapping can be better tailored to the functional anatomy of the area being mapped (See Figure 5). For example, (171) have put forward the idea that language networks consist of functionally specialized “cores” and domain general “periphery.” Interactions between language nodes and with other brain networks are thought to subserve different language functions which may differ depending on task parameters. For example, the word “nail” has different meanings depending on the context of use, and will likely engage different subnetworks when presented as body part, an object, or as an action. This conceptualization of language is consistent with fMRI findings, such as those of Tyler et al. (172), which have shown that the same stimulus can activate different brain regions depending on the context of the task (e.g., different regions are activated when presenting a given object and asking the patient to think of the type of object, the general class of object, or the specific name of the object). It could therefore be useful to design tasks with a single set of stimuli that could be used to potentially activate different brain regions depending upon the broader task demands. On the other hand, cognitive psychological approaches to language are often function- and theory-based. Both cognitive theory and underlying brain mechanisms and understanding of the functional organization of the cerebral cortex will be key, related to the idea of *eliminative materialism*, above. In other words, preceding from neurologically plausible concepts of the organization of language is also critical.

### Beyond Language

To date, much less work has been done on mapping non-language functions—and the notion of non-eloquence in brain areas outside of dominant hemisphere language regions needs to be challenged. The right hemisphere is known to play an important role in visual perception and spatial processing (173, 174), object/face recognition (155, 158, 175–178), socio-emotional processing (7), navigation and learning in a spatial context (179), and attention/neglect (180), and there is great need to develop tasks to assess these functions for the purposes of stimulation mapping.

### Visual-Spatial Processing, Construction, Navigation

Some work exists in the area of spatial processing and navigation with regards to ESM/SEEG, but most represent “one-off” case studies and an occasional targeted experiment rather than a planned effort to study these functions with these technologies. However, deficits in many of these functions can lead to varying degrees of disability for the patient. One example is unilateral spatial neglect, which involves an inability to attend to one side of space (most often the left side with right sided lesions). Line bisection and cancellation tasks are commonly used to assess spatial neglect and discrimination behaviorally, and this function has in turn been mapped to the posterior parietal cortex—more specifically the inferior and superior parietal lobules but also portions of the posterior temporal lobe (139). Mental rotation tasks, where the patient has to determine whether two objects are the same or different by mentally rotating them, can also be used to test the non-dominant parietal lobe (181, 182). Functional MRI studies have implicated bilateral superior parietal, frontal, and inferotemporal cortices during mental rotation with greater non-dominant, right parietal activation often seen (183). Facial perception and line orientation have likewise been tied to the non-dominant parietal lobe (specifically the parietal-occipital junction) as well as the non-dominant posteroinferior frontal lobe (184). Impairment on these tasks tend to contribute to social dysfunction and can impair work performance as well, although this is an area that has never been well-studied.

Navigation has also been studied in humans, but the tasks, physiology and circuits have some dissimilarities with the large knowledge from rodent studies. Firstly, studies of neocortical theta are likely irrelevant to the navigation-related activity that is extensively characterized in rodents. Attention has focused on lower frequency components of the intracranial medial temporal lobe EEG, but while non-hippocampal theta has been reported, in relation to virtual maze tasks up to 8 Hz (185), including in relation to possible grid cells of the entorhinal area (141, 186), hippocampal task-related candidate theta activity has been described at 1–4 Hz and around 8 Hz (140, 187–190) also see (191). Furthermore, periods of enriched theta are very brief (188) and are not coordinated along the human septo-hippocampal axis (192). Given the large number of diverse cortical inputs to the hippocampal formation inferred in humans, spatial tasks may only influence as-yet unidentified subregions of the hippocampus. Similarly, rodent work has called attention to the hippocampal formation, given a role in learning of spatial tasks, but spatial learning and perception obviously involves multiple regions of both neo- and allocortex, again ripe for dissection with task development.

### Executive Control Processes

Monitoring of executive functions is particularly important during frontal resections, but it is important to note that executive functions involve more distributed cortico-cortico and cortico-subcortical networks, and deficits in this domain can develop with damage outside the frontal lobes (193). Executive functions include processes such as planning, shifting from one mental set to another, updating and monitoring of information, problem solving, metacognition, abstract reasoning, and inhibitory control (194). While little work has been done in this area when it comes to cognitive mapping, tasks to consider could include inhibitory control (e.g., Stroop/color-word interference tests, Go/No Go tests), working memory (e.g., reverse digit sequencing), verbal generative fluency (e.g., saying as many words starting with a particular letter within a short period of time) and mental flexibility (e.g., Oral Trail Making Test requiring alternating recitation of ascending numbers and letters). As an example, researchers used SEEG to demonstrate that the supplementary motor area has a role in evaluating the accuracy of actions (147). Similarly, Puglisi et al. (148) used a simplified Stroop paradigm in a ESM setting, and reported that sparing the identified subcortical sites in the non-dominant frontal lobe region led to preserved executive functions as compared to patients who did not previously receive this mapping.

### Learning and Memory

Clinical memory evaluation using ESM and SEEG paradigms has also been minimally explored and is rarely used in clinical practice, although changes in memory comprise some of the worst potential deficits of epilepsy surgical procedures. Estimates of memory decline, which are limited by greater variations in test usage, nevertheless range from 40 to 60% in temporal lobe cases (195–198). These numbers are more modest with minimally invasive surgical procedures but still occur (48, 199). Moreover, we have argued that the current clinical tests of memory are woefully inadequate to fully capture the complexity of memory. Almost all clinically available tasks require the patient to learn solely auditory/verbal or visual content, which they must learn and recall for a very brief period of time. In actuality, human memory is a much more complex construct in which such recall has to be integrated into existing memories (semantic and autobiographical knowledge) and is typically learned in a multimodal fashion (i.e., requires the integration of multisensory and motor input, linguistic/semantic interpretation, etc.) with coding of temporal, spatial, and emotional context. It is possible that we are only testing the most basic of memory subsystems in the process of learning and consolidating information required for effective adaptation to life. Therefore, we need to develop much more advanced measures and test paradigms to allow us to explore these broader aspects of memory. Some of these paradigms can be as simple as combining modalities of learning (e.g., putting a name with a face) and other aspects have only become possible with the advent of technological advances (e.g., virtual reality). Ideally, an array of tasks varying in complexity with dissociable sub-components will allow for the “mental chronometric” process of determining the neural substrates of the basic components (e.g., networks underlying simple encoding of different stimulus modalities) and the broader systems level interactions (e.g., integrating encoded percepts across stimulus modalities, integrating these new memories with existing semantic and autobiographical knowledge bases, processing modulatory feedback from emotional and linguistic systems), which obviously form multiple “scaleable” levels of integrated complexity.

The use of memory paradigms combined with properly designed electrophysiological study (e.g., CCEPs, single unit recordings) could powerfully increase our knowledge of this critical brain function. Recent work with human and non-human primates, for example, is exploring the relationship between the electrophysiology of sleep (e.g., occurrence of sleep spindles) to memory consolidation processes (200–202). Of note, standard clinical neuropsychological batteries only assess patients after time spans under 1 h and never assess patients after periods of sleep (203). This type of work could be carried out in the EMU with intracranially implanted patients, although not without its own set of confounds (e.g., accounting for potential changes in memory related to recent seizure occurrence, changes in antiseizure medications, and a disruptive hospital environment and sleep schedule). Nevertheless, SEEG paradigms with the use of CCEPs is potentially a powerful tool to explore the sub-circuitry of memory processes. George Ojemann carried out a number of memory studies using ESM over the years, and some of his research suggested that the memory system was more complicated than suggested by theoretical models [e.g., episodic memory was disrupted by stimulation of the lateral temporal cortex: (44, 144)]. These findings have more recently been supported by more recent stimulation work through DARPA [i.e., stimulation of lateral TL cortex enhanced memory at some frequencies (146)], and by some initial clinical data involving minimalistic approaches to surgery [lateral TL ablations led to significant verbal memory dysfunction despite preservation of medial TL structures (48)].

Extraoperative single unit recordings can be made when intracranial electrodes are implanted for clinical reasons in patients with refractory epilepsy [see (204)]. While they have provided important insights into memory and cognition, there are a number of limitations. Particularly, it is difficult to continue to record from the same unit for long periods, cell types can be inferred, but not exquisitely defined, and the few neurons that can be recorded, as well as the inability to cover all areas involved in every function (205).

Finally, the possibility of an “*electric Wada*” seems surprisingly absent from the epilepsy surgery landscape. At least, in the case of patients undergoing invasive monitoring with hippocampal electrodes, it is possible to carry out Wada memory paradigms while disrupting subregions and interconnections of the hippocampus and amygdala or even broader structures (145). This seems particularly relevant in this era of minimally invasive surgical procedures as compared to prior surgical epochs where essentially all patients were undergoing procedures that resected multiple regions of the temporal lobe (including much of the temporal pole, anterior inferior and middle temporal gyrus, fusiform, basal temporal lobe, entorhinal/perirhinal cortex, etc.). The Wada is a blunt test in its own right, with many studies suggesting variability in which brain regions are being affected by the delivery of drug [e.g., sodium amobarbital, brevital (206)]. This was less of a problem with open resective surgery but the Wada test may not adequately reflect the potential outcome of a stereotactic laser amygdalophippocampotomy, which primarily affects the amgdalohippocampal complex. Creating an electric Wada appears to be an area where SEEG will be well-suited, but disruptive stimulation will need to be thoughtfully deployed with very low current intensities and careful tailoring based on the stimulation site: Most patients that would be candidates will have medial temporal onset zones—higher frequency stimulation readily causes seizures when applied to the hippocampus and is often avoided (72).

### Social-Emotional Functions

There is increasing recognition of the importance of brain functions beyond cognitive processing, as the preservation of social cognition, emotional processing, and empathy may be equally important in determining quality of life (207), and often represent some of core areas of dysfunction in disorders such as autism and schizophrenia (208, 209). Over the years, socio-emotional tasks (e.g., recognizing emotional state from facial expressions, emotional prosody, self-other distinction, empathy, embodied rotation, and theory of mind tasks) have been shown to depend heavily on the limbic system with significant contributions from the amygdalohippocampal complex, the insula, the cingulate cortex, the anterior temporal lobe (e.g., superior temporal pole), select regions of the broader temporal lobe (e.g., the right temporo-parietal junction), the right dorso-medial prefrontal cortex, the bilateral inferior parietal lobules, and more recently the “default mode network (DMN)” (210–215). The latter is a proposed network derived from functional neuroimaging research that appears to activate whenever an individual is not actively engaged in a task in their environment, and which has been linked to a wide range of cognitive [e.g., forming self-relevant mental constructs, planning for future (216)] and social functions in its own right [e.g., social understanding of others, morality (211, 214)]. These findings relating socio-emotional functions to neural underpinnings were originally formulated from naturally occurring lesion studies in humans and ablation studies in non-human primates (217–221), and have been augmented over the years by data derived from functional neuroimaging studies and to a lesser extent electrophysiological investigation (222, 223). Catani et al. (224) provide a thorough review of the contributors over the past century and a half to these developments, and offer an updated model for the neural substrates underlying emotion, memory, and behavior. These authors reviewed the pioneering work of Papez (225), Yakolev (226), and MacLean (227, 228) among notable contributors, and incorporated the DMN into these earlier models, noting that this network shares neuroanatomical overlap with Papez's circuit. More specifically, the medial aspects of the DMN correspond to the most dorsal aspects of the Papez circuit and are interconnected through the dorsal cingulum. While this is an interesting model, it is worth noting that Papez's circuit was proposed as a mechanism of emotion (225) before the idea of the hippocampus in memory took hold (121), after which Papez's circuit was then recast as a mechanism for memory. Similarly, while ontogeny has some vague relation to the work of MacLean (227), this is a model that has insufficient detail to have explanatory traction in functional localization. Nevertheless, the use of cortical and subcortical mapping paradigms, rarely performed in the thalamus and hypothalamic hamartoma, have not been fully realized as the valuable opportunities that they represent, particularly through the merging of technological advances to create real-life emotional processing situations (e.g., using virtual and augmented reality techniques) that can be coupled with novel data collection methods (e.g., CCEPs, machine learning). In many regards, despite these regions or connections to them being disrupted in many epilepsy surgeries and tumor cases, there has been little clinical attention focused on this likely critical area of function for all of the reasons previously cited.

Theory of mind in particular has emerged as an important facet of social cognition and is one of the few areas that has received some degree of attention in the surgical setting. Critical to this ability—which involves the inference of others' mental/affective states and prediction of behavior(s)—are the posterior inferior frontal gyrus, dorsolateral prefrontal cortex, posterior superior temporal gyrus, and right temporo-parietal junction (207, 229, 230). One such measure that has been adapted to the neurosurgical setting is the *Reading the Mind in the Eyes task* (229), which was developed for use in the autistic population. This task consists of patients matching one of four affective states to 36 serially presented photographs depicting only the eye region of human faces. Use of this novel task has facilitated mapping of theory of mind, and research has shown that patients do not completely recover this ability following pars opercularis resection (231, 232). Additionally, however, we recently had a teenager decline drastically on this task (normal to impaired) who underwent a left stereotactic laser amygdalohippocampotomy only, and whose dysfunction has persisted over time (233). This highlights the need for further study of the regions critical for such functions as well as individual variability that may occur across patients. Finally, mapping of the insular cortex has also led to some interesting disruptions of potential socio-emotional functions, as well as internal perceptions or *interoception*, including pain sensation, interoceptive awareness, emotions, self-recognition, empathy, motivation, craving, alterations in breathing, and time perception (234).

### Consciousness

An ontology of consciousness is developing, with a key and important example of the necessity of a clear ontology in Antti Revonsuo's *Inner Presence* (235). This has helped neuroscience move away from imprecise and all-encompassing notions of consciousness, as well as behavioral approaches to consciousness from clinical neurology, where the level of arousal is a major interest. While important recent work has taken this latter approach to study minimally conscious states, often in the setting of diffuse or at least non-focal brain injury [e.g., (236)], other work has brought us close to examining subject-reported inner experience. Of particular note is the extension of Baars “Global Workspace Theory” from the 1980s [see (237)] to neural substrates and cognitive theory. This approach has informed both cognitive and psychological ideas regarding *phenomenal consciousness* (see Revonsuo) or subjective awareness—a core component of consciousness—such as in the work of Dehaene [e.g., (238)], or in the sphere of SEEG studies, where this has been developed by Naccache et al. (239–246). This work argues, with empirical evidence, that association cortices are fundamental to phenomenal consciousness. Disfunction of these regions in turn leads to a loss of awareness. It is important to note that while phenomenal consciousness may represent a core component of consciousness perhaps its *sine qua non*, there are other elements that are necessary to normal conscious experience that call for mechanistic explanations, such as metacognition, reflection, selfhood, embodiment, autobiographical narrative, introspection, etc. While all of these seem amenable to well-designed studies that might take advantage of the spatial and temporal resolution of SEEG, sparse sampling needs to be overcome, perhaps by combination with functional imaging methods. When rejecting the behaviorist notion that subjective report is not scientific, and accepting that within certain wide bounds, subjectivity is a core and valid type of data [see, for example (238)]. With a better framework for considering the important constituents of consciousness (e.g., wakefulness, perception, attention, multi-modal representation, mnemonic processing, meta-cognitive processes), the time seems right to begin exploring aspects of these mechanisms. To achieve this, new tasks and experimental paradigms are needed.

### Modernization and Standardization of Testing Paradigms and Techniques

**Figure 6:**
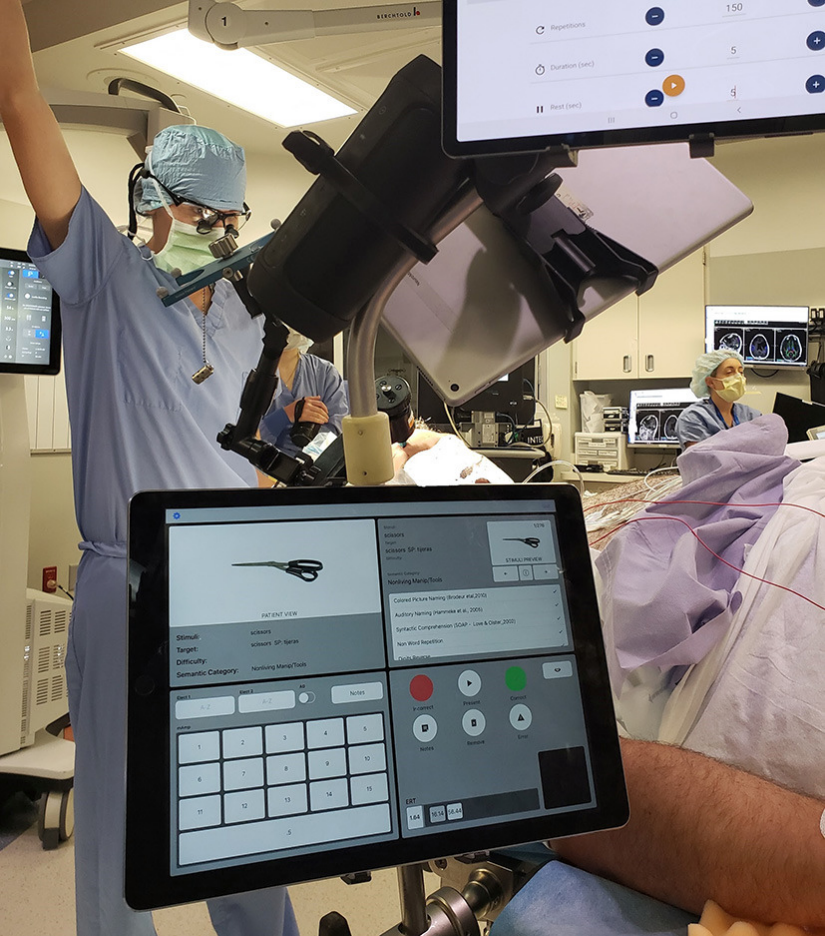
Intraoperative use of the iPad-based Neuromapper tool. Written informed consent was obtained from the individual for the publication of any potentially identifiable images.

It is important to take a highly individualized and functional anatomically informed approach to task selection to optimize mapping/monitoring for any given patient. In addition to greater focus on task development, there is a need to develop effective and innovative methods for administering cognitive testing during ESM more considerate to efficiency and portability of both stimuli and capture methods. Laptop-based systems are widely utilized to display stimuli to patients (e.g., Powerpoint presentations) and allow for extensive stimuli to be stored and displayed electronically but the transition to computers alone does not address the often unstructured and highly variable testing methods used across institutions. Increased consensus in this regard would be valuable in allowing for more comprehensive research endeavors with larger samples across collaborating institutions and further identification of best practices. It would also allow for sharing of common test stimuli, making it possible for more groups to consider more sophisticated mapping paradigms that often take a great deal of preparation time to create. Consensus is becoming increasingly possible through development of specific surgical brain mapping software packages/applications that can be used in a standardized fashion across institutions. One such open-source testing platform is NeuroMapper, which was developed by one of the authors of this manuscript (DSS) (See Figure 6). It is utilized to administer a variety of cognitive paradigms to patients in a highly customized and flexible manner *via* dual iPads, allowing for highly individualized mapping by using baseline performance to select stimulus sets for mapping. This then allows examiners to quickly and easily code patient responses (e.g., correct/incorrect, types of errors made) and monitor changes in task accuracy and reaction times in real time, as well as to obtain video records of patients responses for later review. Another useful feature of NeuroMapper is its portability, given the relatively reduced size and weight of the iPad vs. more traditional laptop/computer setups.

**Figure 7:**
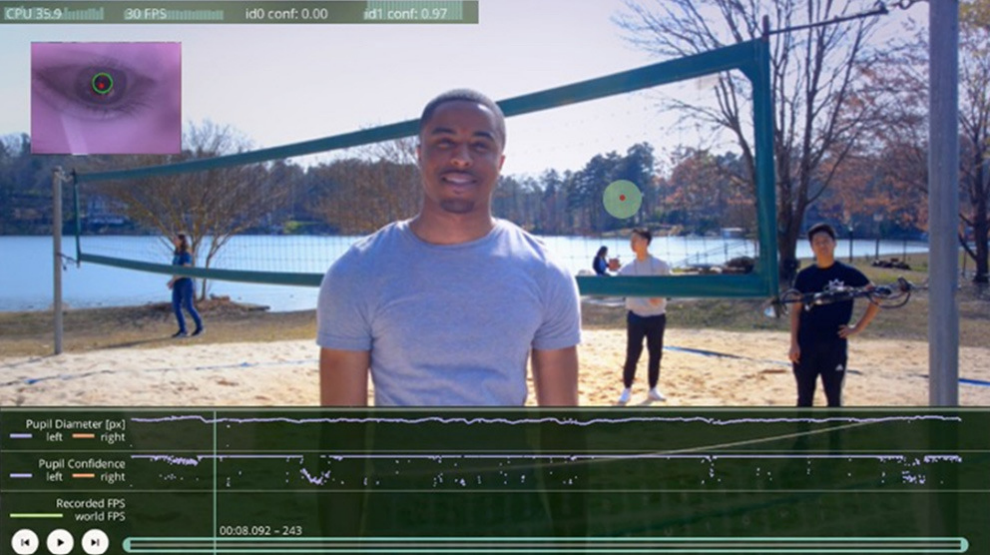
Emory Multimodal Memory Test. A new multimodal tool that is under development for the assessment of multiple domains of cognition and their integration, along with simultaneous recorded eye position and pupil diameter data. Written informed consent was obtained from the individual for the publication of any potentially identifiable images.

We have already suggested many areas of additional test development (e.g., expanded testing of language, adaptation, and creation of tasks of socio-emotional function), but would also like to encourage the exploration of virtual reality and augmented reality techniques to expand the depth and range of constructs that are being examined. These technologies allow for an immersive experience which can be tightly controlled, observed, measured, and manipulated in a manner that allows us to move beyond simple measurement of function yet provides control of the environment while altering task components in a systematic manner. Given the construct of memory, for example, we can continue to test simple episodic recall of a list of words, a story, an object, or a visual scene, but we can also more easily add greater context to the learning, which can pull from semantic (factual information) and autobiographical information (the subject's own personal experiences) and can include both established and novel experiences and information as well. With eye-tracking, motion capture technologies, video-recording, and simultaneous electrophysiological measurement, we can study the patient thoroughly and in a precision manner not previously possible with paper and pencil or even computer-administered tasks. Our own group has created a series of video-vignettes (See Figure 7) with professional actors, which require the patient to learn the content of a brief episodic occurrence in the life of one or more individuals, while learning to recognize those individuals (including face, voice, etc.), the settings in which each event takes place, incidental occurrences in the background, and the interactions between these factors (e.g., “Which person was seen in a given context?” and “What other individuals and objects were present?”). We have created extensive foils that include altering subtle characteristics of each scene and its content to test the limits of recall, including potentially the stylistic manner of information retrieval (e.g., subtasks that can potentially tease out the reliance upon familiarity or recognition or a failure of pattern-separation vs. a failure of language, semantics, or other factors). With current technology and artistic capabilities scenes can be subtly manipulated to allow for the ultimate alternate test forms (e.g., making changes in objects or persons while leaving every other aspect of the scene exactly the same) or to apply eye-tracking mechanisms to determine if changes in repetitive scenes were even noticed based on existing literature on the visual fixation differences for novel vs. old information in patients and controls [a finding observed in human and non-human primates (247, 248)]. These sorts of complex tasks would be ideally suited to a situation in which passive mapping is being used, yet a simplified version (e.g., face-name learning) can be completed using an active stimulation paradigm.

Some examples of the use of virtual reality and electrophysiological studies together already exist both with human, rodents, and non-human primates. Several studies have used virtual reality systems combined with implanted electrodes, for example, to study processes such as spatial navigation in rodents and non-human primates (249, 250). These studies have provided a number of interesting findings, facilitating insight into the neural substrates of allocentric (i.e., a more general, global directional sense) and egocentric navigation (a more local system, based on familiar landmarks and their spatial relationships), and other factors that facilitate or hinder navigation. It appears that the hippocampal formation is critical for allocentric navigation (along with parahippocampal and retrosplenial cortex), but that egocentric processes activate neural systems outside of the medial TL (including medial and posterior parietal lobe regions and caudate nucleus).

**Figure 8:**
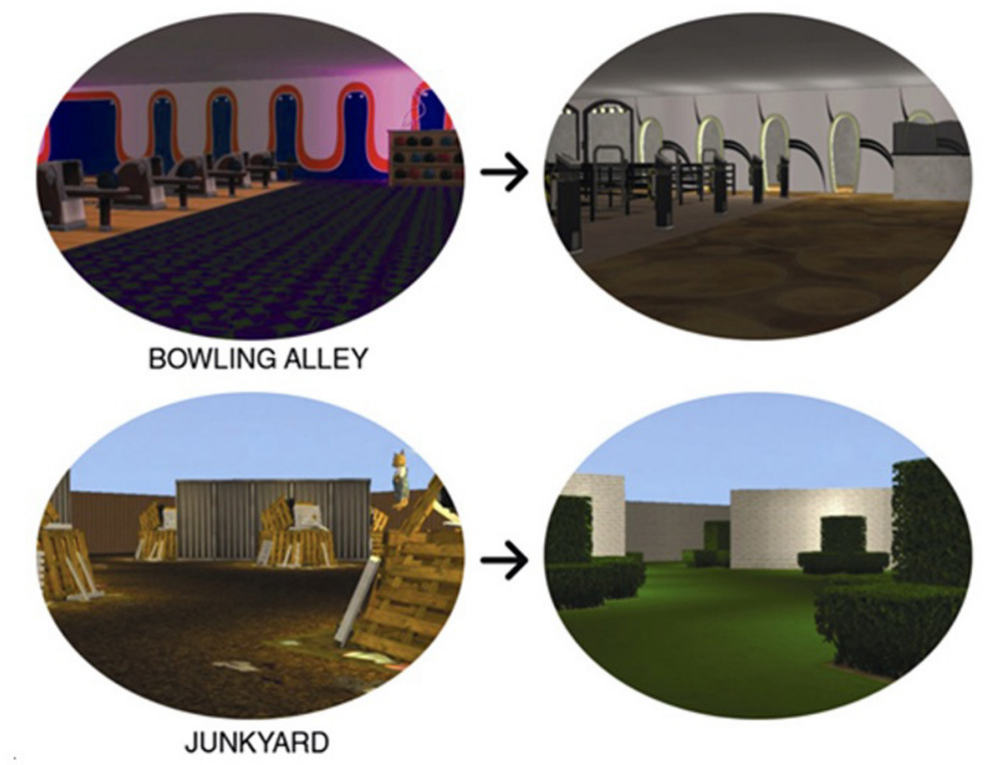
Examining meta-memory with a spatial task. In this virtual reality task from the Cleary Lab (258), subjects can rate feelings of familiarity and deja vu after flying through spatially similar scenes. This is presently being used in the setting of SEEG to examine the anatomy and network activity associated with familiarity. It is also under development with contemporary virtual reality hardware and software.

In humans, a group in France has started piloting the use of a virtual reality headset during awake surgeries in the operating room, using this system to test language but also to explore the ability to understand social gestures (251). There were limitations to what could be accomplished, but they are working on solutions to these issues through ongoing research. Over the last 2 years, virtual tasks have exploded, with ecologically realistic or episodic memory in a shop environment (252, 253), spatial orientation (254), attention (255), and large-scale spatial learning (256). Similarly, standard objects to use in creating these environments (257), and free programming tools (e.g., Unity by Unity Technologies) are making this easier and more standardized (See Figure 8). Combining these rich tasks with intracranial electrophysiology will be difficult, given the complex and unfolding nature of tasks, but much may also be learned. Overall, virtual and augmented reality paradigms will increase our ability to study complex phenomena in a highly controlled manner, and should lead to further insights into the neural substrates of complex behavior of all sorts, which will be helpful for better understanding disease states, navigating neurosurgical procedures to provide optimal benefit with the greatest sparing of function, enhancing our ability to use neuromodulation procedures to treat disease (e.g., seizures, depression) and potentially enhance/restore neurological function, and create the opportunity to develop brain-machine interfaces (259), such as those that have allowed the creation of a bionic prosthetic limb. Of note, all of these “futuristic” advances also call for the need for close collaboration with neuroethicists, discussion between cognitive psychologists and neuroscientists, and potentially input from neurophilosophy, to determine the best path forward in these new frontiers [e.g., (260)].

## Conclusions

Cognitive and emotional mapping with SEEG holds great promise both as a clinical tool and research paradigm for significantly improving our understanding of brain structure-function networks. As laid out in this paper, by augmenting a rich history cortical stimulation mapping and SEEG mapping with advances in neuroimaging (e.g., connectivity metrics; precision volumetrics), neuropsychology/neurophilosophy (e.g., updating old models with advances in brain modeling and theory; adding new measures that tap the rich, complexity of thought and memory), technology (e.g., virtual/augmented reality for humans and non-human primates alike), electrophysiological processing and computational modeling (e.g., CCEPs, machine learning algorithms), the field is poised to make rapid advances. Such potential gains could not only improve the care of patients with brain tumors and epilepsy, but potentially allow us to better understand other neurological diseases (e.g., semantic dementia vs. Alzheimer's disease) while developing more targeted, novel treatments. For example, by understanding the neural circuitry of cognition and emotion and their dysfunction, we may be able to develop more specific drug treatments, better position neuromodulatory devices, or even learn to “relink” damaged pathways and circuits. There is still much exciting “building block” work to be done in each of these subfields, and those of us working with these tools and paradigms should be establishing consortiums to share ideas, resources, and data to enable exponential growth in this field over the next couple of decades.

## Author's Note

This manuscript reflects a review of the relevant published research related to cognitive and behavioral mapping using passive and active stimulation electrophysiological paradigms. It has not been published in any other source and is the original work of the cited authors. DD and NP created an outline for the article, wrote major components, and invited and supervised co-authors with specific expertise in relevant areas to write subsections of the paper. All authors were given an opportunity to edit the manuscript prior to submission for publication.

## Author Contributions

DD and NP planned the paper, created an outline, recruited co-authors to assist with writing specific subsections, wrote several subsections of the paper, and provide editorial oversight. DS, CB, AD, AA, and AK were assigned subsections to write based on their specific expertise, carried out systematic literature searches, and prepared a draft of their assigned section. All co-authors contributed to the editorial process. All authors contributed to the article and approved the submitted version.

## Conflict of Interest

DD receives ongoing funding from Medtronic, Inc. to run a Core Analysis Lab for neuroimaging and cognitive testing in one of their FDA trials, these funds did not contribute in any form to his role in this paper. They were not involved in this paper in any manner, including study design, data collection, analysis, or interpretation, the writing of this article or the decision to submit it for publication. The remaining authors declare that the research was conducted in the absence of any commercial or financial relationships that could be construed as a potential conflict of interest.
